# Matrix M Adjuvanted H5N1 Vaccine Elicits Broadly Neutralizing Antibodies and Neuraminidase Inhibiting Antibodies in Humans That Correlate With *In Vivo* Protection

**DOI:** 10.3389/fimmu.2021.747774

**Published:** 2021-11-23

**Authors:** Fan Zhou, Lena Hansen, Gabriel Pedersen, Gunnveig Grødeland, Rebecca Cox

**Affiliations:** ^1^ Influenza Center, Department of Clinical Science, University of Bergen, Bergen, Norway; ^2^ Center for Vaccine Research, Statens Serum Institut, Copenhagen, Denmark; ^3^ Department of Immunology, University of Oslo and Oslo University Hospital, Oslo, Norway; ^4^ Department of Microbiology, Haukeland University Hospital, Bergen, Norway

**Keywords:** H5N1 (Avian influenza), correlate of protection, adjuvant, Matrix M, pseudotype neutralization, neuraminidase inhibiting antibodies

## Abstract

The highly pathogenic avian influenza H5N1 viruses constantly evolve and give rise to novel variants that have caused widespread zoonotic outbreaks and sporadic human infections. Therefore, vaccines capable of eliciting broadly protective antibody responses are desired and under development. We here investigated the magnitude, kinetics and protective efficacy of the multi-faceted humoral immunity induced by vaccination in healthy adult volunteers with a Matrix M adjuvanted virosomal H5N1 vaccine. Vaccinees were given escalating doses of adjuvanted vaccine (1.5μg, 7.5μg, or 30μg), or a non-adjuvanted vaccine (30μg). An evaluation of sera from vaccinees against pseudotyped viruses covering all (sub)clades isolated from human H5N1 infections demonstrated that the adjuvanted vaccines (7.5μg and 30μg) could elicit rapid and robust increases of broadly cross-neutralizing antibodies against all clades. In addition, the adjuvanted vaccines also induced multifaceted antibody responses including hemagglutinin stalk domain specific, neuraminidase inhibiting, and antibody-dependent cellular cytotoxicity inducing antibodies. The lower adjuvanted dose (1.5µg) showed delayed kinetics, whilst the non-adjuvanted vaccine induced overall lower levels of antibody responses. Importantly, we demonstrate that human sera post vaccination with the adjuvanted (30μg) vaccine provided full protection against a lethal homologous virus challenge in mice. Of note, when combining our data from mice and humans we identified the neutralizing and neuraminidase inhibiting antibody titers as correlates of *in vivo* protection.

## Introduction

Enveloped RNA viruses, such as influenza viruses and coronaviruses, constantly evolve, thus causing zoonotic outbreaks and occasional pandemics in humans. Mutations accumulate over time and enable the virus to escape existing immunity established from previous infection and/or vaccination. This mechanism leads to the emergence of geographic and temporal novel variants, which hamper the effectiveness and efficacy of the vaccines designed based on ancestral viruses. As a result, vaccines targeting enveloped RNA viruses need to be updated at regular intervals. Vaccines capable of inducing broadly cross-protective immune responses are urgently needed.

Since its first isolation in 1996, the highly pathogenic avian influenza (HPAI) H5N1 virus have caused outbreaks in domestic and wild birds worldwide, as well as sporadic animal-human transmissions. To date, 862 human infections have been laboratory confirmed which resulting in 455 deaths (1). Tens of thousands of HPAI H5N1 virus strains have emerged in the last two decades. These variant strains are grouped into 10 clades and dozens of subclades according to the main surface glycoprotein hemagglutinin (HA) gene sequences. All the variants isolated from human infections are from clades 0, 1, 2 and 7 (2–4). To combat the HPAI H5N1 viruses in situations of potential human-to-human transmission, a panel of pre-pandemic H5N1 vaccine candidates from each of the most common (sub)clades have been prepared (3). Different vaccines formats, including subunit, live attenuated, and adenoviral vectores have been tested in clinical trials alone, or in combination with adjuvants such as AS03 and MF59 (5–9). These vaccines elicited protective homologous antibody responses and low to moderate levels of neutralizing antibodies to closely related strains. However, the breadth of cross-neutralizing antibody responses after vaccination has not been fully elucidated.

Compared to the highly variable HA head domain, HA stalk and neuraminidase (NA) are more conserved among circulating strains across different continents and seasons (10–12). Recent studies have revealed functions of non-neutralizing antibodies targeting these more conserved domains. For example, HA stalk specific antibodies can block viral genome release into the cytoplasm; whilst NA specific antibodies reduce progeny virion release from infected cells (13). In addition, non-neutralizing antibodies can trigger cytotoxicity and phagocytosis to clear infected cells (14, 15). However, whether these non-neutralizing antibodies correlate with *in vivo* protection against the highly pathogenic H5N1 virus remains unclear.

We have conducted a clinical trial with a virosomal H5N1 vaccine with Matrix M adjuvant in 60 adults. We have previously demonstrated that the adjuvanted H5N1 vaccines elicited potent vaccine specific neutralizing antibodies, and to a lesser extent cross-reactive hemagglutination inhibition (HI) antibodies and Th1 and Th2 CD4+ T cell responses against closely related strains (16–18). Here, we established an expanded panel of H5N1 pseudotypes covering all (sub)clades isolated from human infections; and characterized the kinetics and breadth of antibody responses after vaccination, including dissection of the multifaceted non-neutralizing antibody responses. We also assessed the *in vivo* protection from vaccine induced antibodies in a passive transfer murine model and investigated immunological candidates for correlates of protection.

## Material and Methods

### Study Design

Sixty healthy adult volunteers (20-49 years old) were enrolled in an open label phase I dose escalating clinical trial early 2009 at Haukeland University Hospital, Bergen, Norway (www.clinicaltrials.gov, NCT00868218) (16). The study was approved by the Regional Committee for Medical Research Ethics, Northern Norway and the Norwegian Medicines Agency. All participants provided written informed consent before inclusion into the study.

Participants were randomized into 4 groups, and intramuscularly vaccinated twice with the H5N1 virosomal vaccine (Crucell Berna Biotech) containing 30μg HA alone, or 1.5, 7.5 or 30μg HA with 50μg Matrix M adjuvant (Novavax) at 3-week interval (16). None of the participants had previously received an H5N1 vaccine.

### Vaccine and Sampling

A monovalent inactivated virosomal H5N1 vaccine, containing vaccine strain NIBRG-14, a virus derived from A/Vietnam/1194/2004 (H5N1) and A/Puerto Rico/8/1934 (H1N1) using reverse genetics, was used in the clinical trial. The influenza surface antigens HA and NA were purified from beta-propiolactone (BPL) inactivated egg grown viruses, mixed with lecithin and incorporated into the phospholipid bilayer by spontaneous formation of the virosomes. The HA content of the vaccine was quantified by single radial diffusion, and the presence of NA was confirmed.

The adjuvant Matrix M used in the trial was the 3^rd^ generation immune stimulating complex, which contains Matrix-A and Matrix-C fractions produced from purified *Quillaja* saponin fractions A and C, at the proportion of 91:9. The vaccine was formulated as 30μg HA alone, or 1.5, 7.5 or 30μg HA with 50μg Matrix M adjuvant, filled into single use syringes, and stored at 4°C until use. All participants (n=60) received 2 doses of the vaccine at a 3-week interval, except one withdrawal from the 7.5µg HA adjuvanted vaccine group.

Blood samples were collected before, and up to 42 days after vaccination. Sera were separated, aliquoted and stored at -80°C until use.

### Hemagglutination Inhibition Assay

All sera were treated with receptor-destroying enzyme (RDE, Seiken) at a ratio of 1 in 4 at 37°C for 18h, and heat treated at 56°C for 1h. The treated sera were analysed in duplicate (2-fold serial dilution, starting from 1:10) with 4 hemagglutinating units of viruses and 0.8% horse red blood cells, as previously described (16, 17). A panel of reassortant H5N1 viruses were used, including A/Vietnam/1194/2004 (NIBRG-14, vaccine strain, clade 1), A/Indonesia/5/2005 (IBCDC-RG2, subclade 2.1.3.2), A/turkey/Turkey/1/2005 (NIBRG-23, subclade 2.2.1), and A/Cambodia/R0405050/2007 (NIBRG-88, subclade 1.1). The hemagglutination inhibition (HI) titer was determined as the reciprocal of the highest sera dilution giving 50% inhibition of hemagglutination. Non-detected samples were assigned a value of 4 for calculation purpose.

### Pseudotype-Based Neutralization Assay

H5N1 pseudotypes were generated by co-transfecting lentiviral vectors pHR’CMV-Luc, pCMVRΔ8.2, pCMVR-H5HA, and pCMVR-N1NA into HEK293T cells as previously described (19, 20). A panel of pCMVR-HA constructs encoding H5HA from A/Vietnam/1203/2004 (Vietnam, clade 1), A/Indonesia/5/2005 (Indonesia, subclade 2.1.3.2), A/Turkey/65596/2006 (Turkey, subclade 2.2.1), A/common magpie/Hong Kong/5052/2007 (HK5052, subclade 2.3.2.1), A/Shenzhen/406H/2006 (Shenzhen, subclade 2.3.4), A/Shanxi/2/2006 (Shanxi, clade 7), and pCMVR-N1NA construct encoding NA from A/Thailand/(KAN-1)/2004 were used to prepare H5N1 pseudotypes.

All sera were heat inactivated and analysed in duplicate (4-fold serial dilution, starting from 1:10) with H5N1 pseudotypes corresponding to 20,000 to 200,000 relative luciferase activity (RLA), as previously described (20). The pseudotype-based neutralization (PN) titer (IC_80_) was determined as the reciprocal of the sera dilution giving 80% reduction of RLA. PN titers were normalized based on HIV-1 gag p24 quantities for different pseudotypes. Non-detected samples were assigned a value of 2 for calculation purpose.

### Enzyme-Linked Immunosorbent Assay

H5HA stalk and N1NA specific immunoglobulin G (IgG) were quantified in sera using the enzyme-linked immunosorbent assay (ELISA) developed in-house, as previously described (21). Serially diluted sera were analysed in Maxi Sorp 96-well plates coated with 1 μg/ml recombinant chimeric HA (cH9/5HA) that combines the H5HA stalk domain from NIBRG-14 strain with an HA globular head domain from A/guinea fowl/Hong Kong/WF10/1999 H9 influenza A virus, or 1 μg/ml recombinant N1 neuraminidase. Immunoglobulin concentrations were interpolated from standard human IgG curves. For calculation purposes, non-detected samples were assigned as 0.04 μg/ml against cH9/5HA and 0.005 μg/ml against N1NA.

### Virus Neutralization Assay

All sera were heat inactivated and analysed in duplicate (2-fold serial dilution, starting from 1:10) with 100 TCID_50_ reassortant cH9/1N3 virus in MDCK cells, as previously described (22). The cH9/1N3 virus contains the HA stalk domain from A/California/07/2009 H1 strain, an HA globular head domain from A/guinea fowl/Hong Kong/WF10/1999 H9 strain, and N3NA from A/swine/Missouri/4296424/2006 virus. The virus neutralization (VN) titer was measured with 0.7% turkey red blood cells and determined as the highest serum dilution giving complete hemagglutination. Non-detected samples were assigned a value of 5 for calculation purpose.

### Enzyme-Linked Lectin Assay

All sera were treated at 56°C for 45min and analysed in duplicate (3-fold serial dilution, starting from 1:5) with a reassortant NIBRG-73 virus, as previously described (22). The NIBRG-73 virus has N1NA from the NIBRG-14 strain and H7HA from A/equine/Prague/1956 virus. The neuraminidase enzymatic activity was measured with fetuin, horseradish peroxidase-conjugated peanut agglutinin and o-phenylenediamine dihydrochloride (Sigma-Aldrich), and read as optical density (OD) value at 490nm. The neuraminidase inhibition (NI) titer (IC_50_) was calculated as the reciprocal dilution of sera giving 50% reduction in enzymatic activity. Non-detected samples were assigned a value of 2 for calculation purpose.

### Antibody-Dependent Cellular Cytotoxicity Reporter Assay

All sera were heat inactivated, serially diluted and analysed using ADCC Reporter Bioassay kit (Promega), as previously described (22). MDCK cells infected with NIBRG-14 virus at multiplicities of infection (MOI) 0.34 were used as target cells, and Jurkat/NFAT-luc cells were used as effect cells. The antibody-dependent cellular cytotoxicity (ADCC) reporter activity was measured with Bio-Glo Luciferase Assay Reagent (Promega) as relative luciferase activity (RLA). ADCC titer (EC_50_) was calculated as the reciprocal dilution of sera giving 50% of maximum RLA. Non-detected samples were assigned a value of 2 for calculation purpose.

### Passive Transfer and Viral Challenge in Mice

Six to eight weeks old female BALB/c mice (Taconic, Denmark) were housed under specific-pathogen free conditions at Rikshospitalet, Oslo University Hospital. All animal experiments were approved by the Norwegian Food Safety Authority.

Pooled human sera or saline (400µl/mouse) were injected intraperitoneally (i.p.) into mice (n=12/group) 1 day prior to viral challenge. Mice were anaesthetized by subcutaneous (s.c.) injection of Hypnorm/Dormicum (0.05ml working solution/10g) and infected intranasally (i.n.) with 5 MLD_50_ of NIBRG-14 virus in 20 µl/mouse (10µl/nostril). Mice were monitored for survival and weight loss for 2 weeks after challenge, with an endpoint of 20% weight reduction, as required by the Norwegian Food Safety Authority. Mice that lost more than 20% body weight were euthanized by cervical dislocation. For mice that did not lose weight during the 2-week monitoring, the maximum body weight loss was assigned as 0.01% for calculation purpose. At days 3 and 5 post challenge, both lobes of lungs were harvested (n=3 mice/group), snap frozen and stored at -80°C until use.

### Virus Quantification

Frozen lung tissues were weighted and homogenized in DMEM with 1% antibiotics. NIBRG-14 virus was quantified in TCID_50_ on MDCK cells (23). Hemagglutination assay with 0.7% human red blood cells was used to measure the viral load. The limit of detection was 22.49 TCID_50_/ml of homogenate.

### Phylogenetic Tree

Full-length HA protein amino acid sequences from influenza type A viruses were downloaded from NCBI Influenza Virus Database. Phylogenetic analyses were performed at ngPhylogeny.fr using MAFFT (Multiple Alignment using Fast Fourier Transform, default settings), BMGE (Block Mapping and Gathering with Entropy, default settings), and PhyML (Phylogeny software based on the Maximum-likelihood, default settings) (24, 25). See **Supplementary Table 1** for the accession no. of all HA amino acid sequences used.

### Statistical Analyses

Biological replicates were used in all experiments, unless otherwise stated. Antibody quantification results including HI, PN, VN, NI, ADCC titers, and IgG concentrations, and lung viral load results were Ln transformed prior to statistical tests. Turkey’s multiple comparisons and Fisher’s LSD test were performed in two-way analysis of variance (ANOVA). False Discovery Rate controlled multiple comparisons were performed in Nonparametric Kruskal-Wallis test. Nonparametric Spearman correlations and Pearson correlations were tested, linear fitting curves were plotted when Spearman or Pearson P <0.10. In multiple linear regression analyses, all independent variables were centered prior to test. * P<0.05, ** P<0.01, *** P<0.001, all P values are two-tailed. All statistical analyses were performed with GraphPad Prism 7 and SPSS 25.

## Results

### Study Design

**Figure 1:**
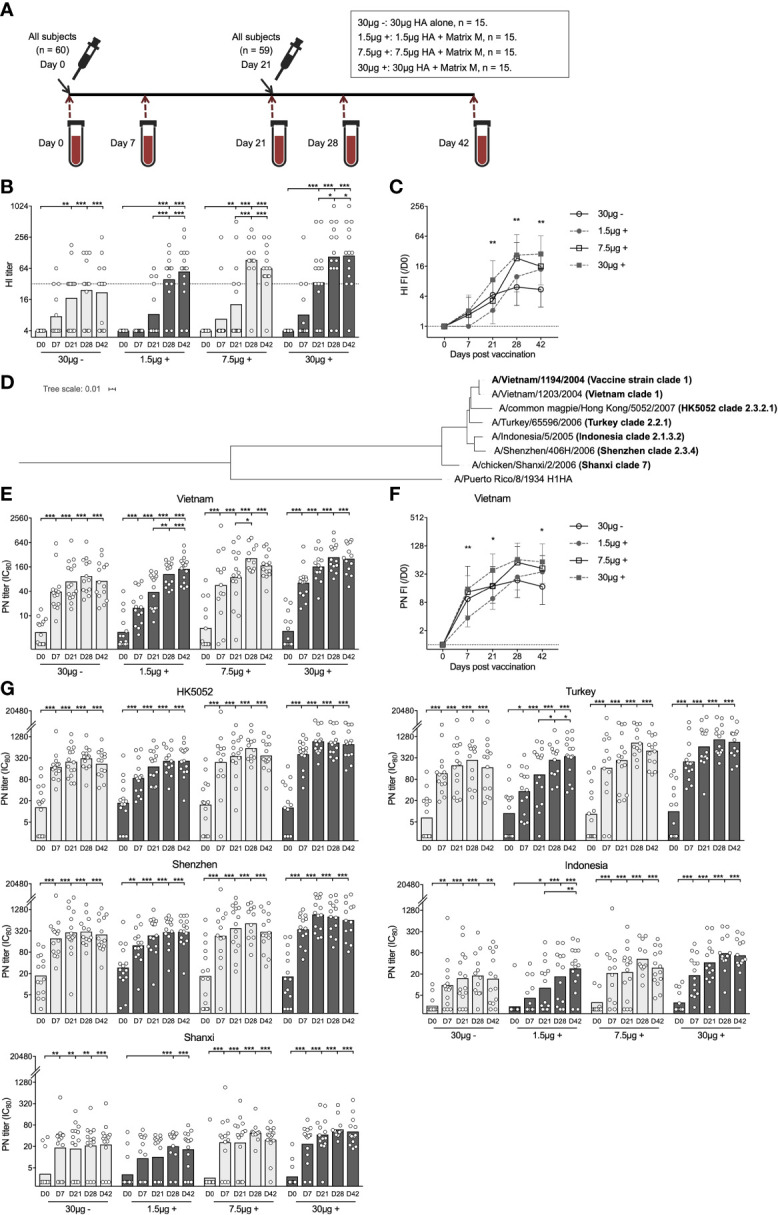
Adjuvanted H5N1 vaccine rapidly elicited strain specific and cross-reactive neutralizing antibody responses. **(A)** An Illustration of the study design. Sixty adults (20 to 49 years old) were enrolled in this study and vaccinated with inactivated virosomal H5N1 vaccine (Crucell| Berna Biotech, Switzerland). The adults were randomized into 4 groups of 15 subjects and received two doses of H5N1 vaccine with 30 μg hemagglutinin (HA) alone (30μg -), or 1.5, 7.5 and 30 μg HA adjuvanted with Matrix M (1.5 μg +, 7.5 μg +, and 30 μg +, respectively), at an interval of 21 days. Serum samples pre- (day 0), 7 days, 21 days, 28 days, and 42 days post-vaccination were collected. One subject withdrew after first dose vaccine in 7.5μg + group. **(B, C)** Hemagglutination inhibiting titer (HI titer, **B**) and fold-induction (FI) after vaccination (HI FI,/D0, **C**) was measured against NIBRG-14 virus (vaccine strain). **(D)** Phylogenetic tree shows the genetic divergence between H5HA from the vaccine strain and heterologous H5HAs tested in pseudotype-based neutralization (PN) assay. H1HA from A/Puerto Rico/8/1934 was used as a reference. Phylogenetic analyses were performed at ngPhylogeny.fr. **(E, F)** Vaccine specific neutralizing antibodies were measured in PN assay against pseudotyped virus derived from A/Vietnam/1203/2004 virus (Vietnam). The PN titer (IC_80_, **E**) was calculated as the reciprocal dilutions of the sera that gave 80% inhibition. Fold-induction after vaccination (PN FI,/D0, **F**) is shown. **(G)** Cross-neutralizing antibody titers (IC_80_) were measured against 5 strains of pseudotypes expressing heterologous H5HAs listed in D All antibody responses were measured using serum samples before (D0) and after vaccination (D7, D21, D28, and D42). The geometric mean values are shown as bars, and each symbol represents one subject **(B, E, G)**. The geometric mean of fold-inductions in each group ± geometric standard deviation as error bar is shown **(C, F)**. *P < 0.05, **P < 0.01, ***P < 0.001 (Antibody titers and fold-inductions were Ln transformed in statistical analyses. Turkey’s multiple comparisons between pre-prime (D0) and post-prime (D7, D21, D28, D42), and between pre-boost (D21) and post-boost (D28 and D42) in each group were performed in two-way ANOVA in **(B, E, G)** Turkey’s multiple comparisons between 30 μg + and 1.5 μg + after prime (D7 and D21), and between 30 μg + and 30 μg - after boost (D28 and D42) were performed in two-way ANOVA in **C, F**). The horizontal dotted lines indicate HI titer of 32 **(B)**, and fold-induction of 1 **(C, F)**. Duplicates were performed in all experiments.

Sixty adults were divided into 4 groups and vaccinated with the H5N1 virosomal vaccine alone or escalating doses of adjuvanted vaccine: Group 30μg - (30μg HA alone, 15 subjects), Group 1.5μg + (1.5μg HA with adjuvant Matrix M, 15 subjects), Group 7.5μg + (7.5μg HA with adjuvant Matrix M, 15 subjects), and Group 30μg + (30μg HA with adjuvant Matrix M, 15 subjects). All subjects received a boosting dose 3 weeks after the first priming dose, except for one withdrawal after the first dose in 7.5μg + group (**Figure 1A**). Twenty-five subjects had earlier received seasonal influenza vaccine(s) or reported influenza virus infection (**Table 1**). Sequential pre- and post-vaccination serum samples up to 42 days were collected from all vaccinees.

**Table 1:** The demographics of the subjects enrolled in the study.

Group(Vaccine administered^1^)	Total(N/A^2^)	30μg - (30μg HA)	1.5μg +(1.5μg HA adjuvanted with 50μg Matrix M)	7.5μg +(7.5μg HA adjuvanted with 50μg Matrix M)	30μg +(30μg HA adjuvanted with 50μg Matrix M)
No. of subjects	60	15	15	15^3^	15
Gender, M/F	22/38	6/9	5/10	7/8	4/11
Median age, years (Range)	30 (20-49)	30 (20-41)	26 (21-44)	28 (22-42)	30 (25-49)
No. of subjects with previous vaccinations and/or infection^4^	25	6	8	4	7

^1^The vaccine was supplied as prefilled syringes of pre-formulated virosomal vaccine with Matrix M.

^2^Not applicable (N/A).

^3^One withdrawal after first dose vaccine in 7.5μg + group.

^4^Annual seasonal influenza vaccines during 2005/06-2008/09 seasons were included as previous vaccinations. Influenza infection during 2008/09 season was included as infection. In addition, three subjects received the H7N1 vaccine in a clinical trial in 2006, which was not included in the table.

### Adjuvanted H5N1 Vaccines Rapidly Elicits Strain Specific and Broadly Cross-Clades Neutralizing Antibodies

We assessed HA specific antibodies using the HI assay. None of the vaccinees had detectable antibodies before vaccination. After priming, only the 30μg + group showed potent increases of vaccine specific antibodies. After boosting, all 3 adjuvanted groups had antibodies above the protective level (HI titer ≥32), while the non-adjuvanted 30μg - group remained below the protective level (**Figure 1B**). The 1.5μg + group had significantly lower antibody fold-induction after priming compared to the 30μg + group, while after boosting the difference diminished. On the contrary, the 30μg - group showed significant antibody induction after priming but limited further increase after boosting (**Figure 1C**).

We further analysed antibody responses using pseudotype-based neutralization (PN) assay. In 24 subjects, low levels of pre-existing vaccine specific neutralizing antibodies were detected. Of note, the vaccine elicited potent homologous antibody responses 7 days after priming in all 4 groups, which were further significantly elevated to higher level in the 1.5μg + and 7.5μg + groups after boosting. The 1.5μg + group had lower antibody fold-induction after priming compared to the 30μg + group but reached similar neutralizing antibody titer after boosting; whilst the 30μg - group had rapid antibody increase 7 days after priming but no significant boost after the second dose (**Figures 1E, F**).

To explore the breadth of the vaccine elicited cross-neutralizing antibodies, we developed a panel of H5N1 pseudotypes expressing divergent H5 hemagglutinins covering multiple (sub)clades of H5N1 viruses isolated from humans (**Figure 1D** and **Supplementary Table 1**). Remarkably, cross-neutralizing antibodies were elicited to equivalent titers as compared to vaccine specific antibodies in all 4 groups when tested against the 3 closely related pseudotypes, namely HK5052 (subclade 2.3.2.1), Turkey (subclade 2.2.1) and Shenzhen (subclade 2.3.4). Meanwhile, although significantly boosted by vaccination, lower levels of cross-neutralizing antibodies were detected to the 2 more distant pseudotypes: Indonesia (subclade 2.1.3.2) and Shanxi (clade 7). Nevertheless, broadly neutralizing antibodies were detected in all the subjects in the 30μg + group across all the (sub)clades (**Figure 1G**).

### Multifaceted Antibody Responses After H5N1 Vaccines

**Figure 2:**
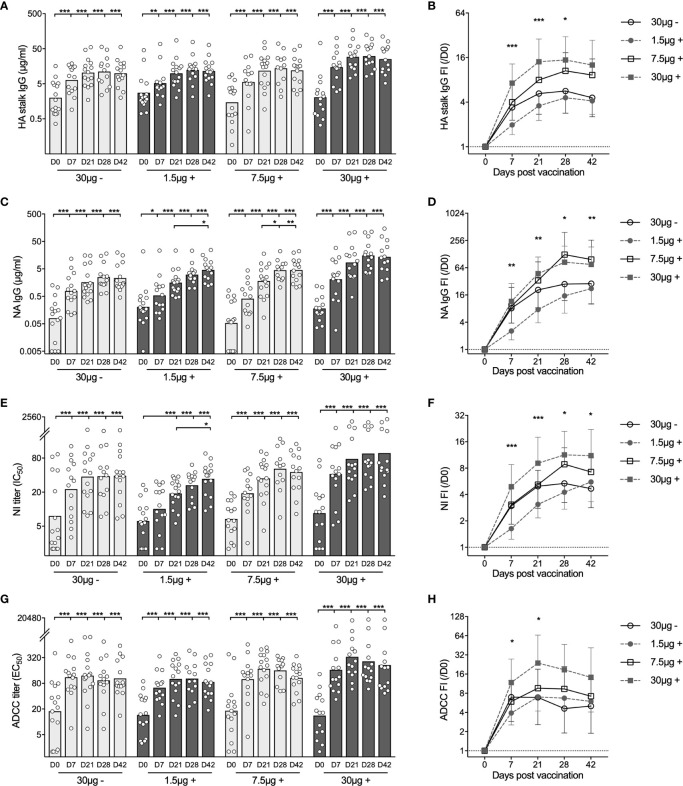
Adjuvanted H5N1 vaccines elicit multifaceted antibody responses. **(A, B)** H5HA stalk specific antibodies were measured against the recombinant chimeric protein cH9/5HA, which consists of HA stalk domain from H5 A/Vietnam/1203/2004 strain and irrelevant H9HA head domain. Concentrations of total H5HA stalk specific antibody measured in ELISA **(A)** and fold-induction (FI) after vaccination (IgG FI,/D0, **B**) are shown. **(C, D)** Total neuraminidase (NA) specific antibodies were measured in ELISA against N1NA from A/Vietnam/1203/2004 virus. Antibody concentrations **(C)** and fold-induction after vaccination (IgG FI,/D0, **D**) are shown. **(E, F)** Pre- and post-vaccination sera were tested for inhibiting NA enzymatic activity in ELLA against reassortant NIBRG-73 virus, which expresses NA from H5N1 vaccine strain and irrelevant H7HA. The neuraminidase enzymatic activity inhibition titer (NI IC50, **E**) was calculated as the reciprocal dilutions of the sera that gave 50% inhibition. Fold-induction after vaccination (NI FI,/D0, **F**) is shown. **(G, H)** Sera were tested for inducing antibody-dependent cellular cytotoxicity (ADCC) in ADCC Reporter Bioassay against the vaccine strain (NIBRG-14). The ADCC titer (EC50, **G**) was calculated as the reciprocal dilutions of the sera that gave 50% of the maximal biological response. Fold-induction after vaccination (ADCC FI,/D0, **H**) is shown. The geometric mean titers are shown as bars, and each symbol represents one subject **(A, C, E, G)**. The geometric means of fold-induction in each group ± geometric standard deviation as error bar are shown **(B, D, F, H)**. *P < 0.05, **P < 0.01, ***P < 0.001 (Antibody titers and fold-inductions were Ln transformed in statistical analyses. Turkey’s multiple comparisons between pre-prime (D0) and post-prime (D7, D21, D28, D42), and between pre-boost (D21) and post-boost (D28 and D42) in each group were performed in two-way ANOVA in **A, C, E, G.** Turkey’s multiple comparisons between 30 μg + and 1.5 μg + after prime (D7 and D21), and between 30 μg + and 30 μg - after boost (D28 and D42) were performed in two-way ANOVA in **B, D, F, H**). The horizontal dotted lines indicate fold-induction of 1 **(B, D, F, H)**. Duplicates were performed in all experiments.

Total HA stalk specific antibodies were measured in ELISA against the recombinant chimeric protein cH9/5HA, which consists of the HA stalk domain from the vaccine strain and an irrelevant H9HA head. All subjects were found to have pre-existing HA stalk specific antibodies. As early as 7 days after priming, we observed significant antibody increases in all 4 groups. The 30μg + group had significantly higher antibody fold-induction as compared to the 1.5μg + and 30μg - groups (**Figures 2A, B**).

We next assessed neuraminidase (NA) specific antibodies by ELISA (**Figures 2C, D**). In 51 subjects, NA specific antibodies were detected prior to vaccination. The H5N1 vaccines elicited potent antibody increases in all 4 groups at 7 days after priming. Further significant antibody increases were found after boosting in 1.5μg + and 7.5μg + H5N1 groups (**Figure 2C**). Overall, the 7.5μg + and 30μg + groups showed equivalently potent responses, while the 1.5μg + and 30μg - groups had lower fold change of NA specific antibodies (**Figure 2D**).

To quantify the antibodies that inhibit NA enzymatic activity, we performed enzyme-linked lectin assay (ELLA). Before vaccination, detectable NA inhibition (NI) titers were observed in 36 of 60 subjects. Vaccinees in the groups 30μg -, 7.5μg + and 30μg + had significant antibody increases 7 days after priming. While vaccinees in the 1.5μg+ group showed antibody induction 21 days after priming, which increased significantly after boosting (**Figure 2E**). The kinetics of NI antibody fold change among 4 vaccine groups was similar to the kinetics of NA specific binding antibodies by ELISA (**Figures 2D, F**).

Lastly, we measured ADCC inducing antibodies. Thirty-seven out of 60 subjects had pre-existing ADCC inducing antibodies. The H5N1 vaccines further elevated the antibody levels, which remained high after boosting in all groups, especially the 30μg+ group (**Figures 2G, H**).

In summary, the virosomal H5N1 vaccines elicited potent and multifaceted antibody responses. The adjuvanted intermediate (7.5μg +) and high (30μg +) dose vaccines potently induced antibody increases 7 days after priming, which were further elevated or maintained after boosting. By comparison, the adjuvanted low (1.5μg +) dose vaccine showed delayed antibody kinetics; whilst the non-adjuvanted (30μg -) vaccine elicited antibodies of an overall lower magnitude.

### H5N1 Vaccines Induced Robust Antibody Responses

**Figure 3:**
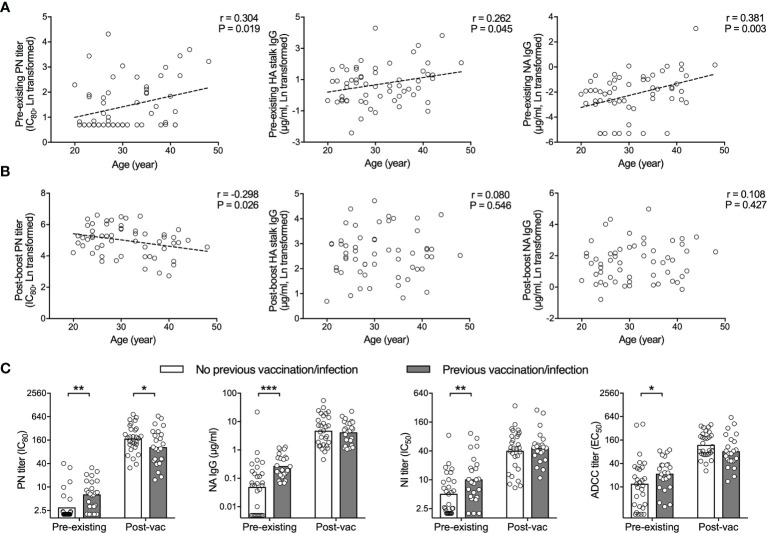
H5N1 vaccines elicit potent antibody responses regardless of pre-existing humoral immunity. **(A, B)** HA specific neutralizing antibodies (PN titer, left), total HA stalk specific antibodies (IgG, center) and total NA specific antibodies (IgG, right) tested in pre-vaccination sera (pre-existing, D0, **A**) correlate with age. Inverse or no significant correlation with age is found in sera after vaccination (post-vac, D42, **B**). **(C)** Subjects with previous vaccination and/or infection had significantly higher pre-existing antibodies, compared to subjects without previous vaccination or infection. After vaccination, the difference was abolished. HA specific neutralizing antibodies (PN titer, far left), total NA specific antibodies (IgG, central left), neuraminidase enzymatic activity inhibiting antibodies (NI titer, central right), and antibody-dependent cell-mediated cytotoxicity (ADCC) inducing antibodies (ADCC titer, far right) were tested in sera before (pre-existing) and 42 days after vaccination (post-vac). The geometric mean titers are shown as bars **(C)**, and each symbol represents one subject **(A–C)**. Linear fitting curve was plotted as dotted line when Pearson correlation P < 0,05. Pearson r and P values are noted for each correlation **(A, B)**. *P < 0.05, **P < 0.01, ***P < 0.001 (Antibody titers and concentrations were Ln transformed in statistical analyses. Fisher’s LSD test between subjects with previous vaccination/infection and subjects without was performed in two-way ANOVA in **(C)**.

As the majority of our subjects had some detectable antibodies against the H5N1 vaccine components prior to vaccination, we investigated whether pre-existing immunity hampers the H5N1 vaccine elicited antibody responses. Firstly, the age of the subjects enrolled in this study ranged from 20 to 49 years old (**Table 1**). We saw significant correlations between the age and pre-existing level of neutralizing antibodies, HA stalk and NA specific binding antibodies (**Figure 3A**). In contrast, the antibody levels after H5N1 vaccination had inverse or no correlation with age (**Figure 3B**). Next, subjects who had had seasonal influenza vaccination or infection within 5 years prior to the current study showed significantly higher levels of pre-existing neutralizing, NA specific and ADCC inducing antibodies. H5N1 vaccination boosted antibodies to comparably high levels in all subjects (**Figure 3C**). Collectively, the H5N1 vaccines induced robust and multifaceted antibody responses regardless of age, vaccination/infection history or pre-existing antibody levels.

### Human Immune Post H5N1 Vaccination Sera Confer *In Vivo* Protection

**Figure 4:**
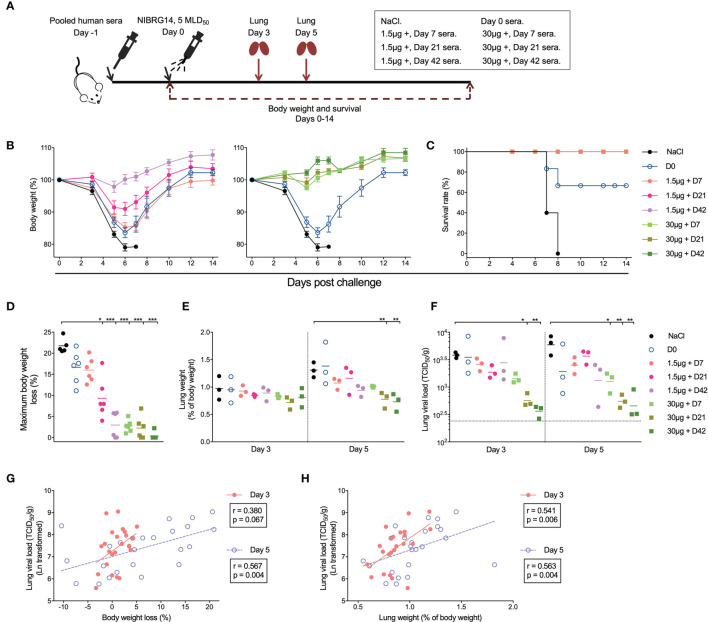
Adjuvanted H5N1 vaccines elicit antibodies provided *in vivo* protection against lethal virus challenge. **(A)** Illustration of the experiment set-up for serum transfer and virus challenge. Pre-vaccination sera from subjects in 1.5μg + and 30μg + groups were pooled together as D0 sera. Sera from days 7, 21 and 42 after vaccination from subjects in 1.5μg + or 30μg + groups were pooled separately. Group-and time-point-wise pooled sera were administered intraperitoneally to female BALB/c mice (n=12 per group). NaCl was given as control. One day later, the mice were infected intranasally with 5× 50% mice lethal dose (MLD50) of NIBRG-14 virus. Body weight and survival were monitored for 14 days after challenge. Lungs from 3 mice per group were collected 3 and 5 days after infection for weight and viral load measurement. **(B, C)** The body weight loss **(B)** and survival rate **(C)** of the mice in different groups are shown. **(D)** Maximum body weight loss was calculated for each individual mouse as the maximum body weight loss through 14 days monitoring, which occurred at 5, 6 or 7 days after challenge. For mice with no body weight loss observed through monitoring, maximum weight loss was assigned as 0,01%. **(E, F)** The lung weight of the 3 mice sacrificed on days 3 and 5 after virus challenge was measured and is shown as the mean of percentage of pre-infection body weight (% of body weight, **E**). The viral load of NIBRG-14 virus was measured in MDCK cells as the reciprocal dilutions of lung homogenates that gave 50% tissue culture infection dose (TCID50) and standardized based on the lung weight (TCID50/g, **F**). The dotted line indicates the lowest detectable viral load in the assay. **(G, H)** The standardized lung viral load (TCID50/g) 3 and 5 days after infection correlate with the body weight loss **(G)** and the lung weight **(H)**. The body weight of mice in each group is shown as mean ± standard deviation as error bar **(B)**. Means **(D, E)** and geometric means **(F)** are shown as horizontal lines, and each symbol represents one mouse **(D–H)**. *P < 0.05, **P < 0.01, ***P < 0.001 (lung viral load was Ln transformed in statistical analyses. False Discovery Rate controlled multiple comparisons between mice receiving NaCl and pooled sera were performed in Nonparametric Kruskal-Wallis test in **D–F**). The standardized lung viral load was Ln transformed in statistical analyses. Linear fitting curve is plotted as solid line (Day 3) or dotted line (Day 5) when nonparametric Spearman P < 0.10. Spearman r and P values are noted for each correlation **(G, H)**. Duplicates were performed in lung weight and viral load measurement.

To study whether the H5N1 vaccine induced antibodies are protective against infection, we performed passive serum transfer and virus challenge in a murine model. Briefly, we pooled pre-vaccination sera from groups 1.5μg + and 30μg + as D0 sera, and post-vaccination sera were pooled within each group from all vaccinees in the 1.5μg + and 30μg + groups for days 7, 21 and 42. Pooled immune sera or saline were then transferred into mice one day prior to a viral challenge (5×MLD_50_) with the NIBRG-14 virus. Body weight was monitored for 14 days after challenge (**Figure 4A**). Mice that received saline or D0 sera became ill and rapidly lost weight. All 5 mice receiving saline and 2 out of 6 mice receiving D0 sera died between days 7 and 8 after challenge. By contrast, all mice that received post-vaccination sera survived. Mice that received sera 7 and 21 days post-vaccination from group 1.5μg + showed mild symptoms and lost a maximum of 16% and 9.3% of body weight, respectively, although they quickly recovered. Mice receiving day 42 sera from the group 1.5μg + and sera at all 3 time points from the group 30μg + displayed no symptoms of disease and had < 3% body weight loss at maximum (**Figures 4B–D**).

We measured the lung weight and viral load at 3 and 5 days after challenge. Mice that received days 21 and 42 sera from group 30 μg + showed lower lung weight at 5 days post challenge, and lower viral loads at days 3 and 5, compared to the mice receiving saline (**Figures 4E, F**). In addition, we observed good correlations between the lung viral load and body weight loss, as well as between the lung viral load and the lung weight (**Figures 4G, H**). To summarise, immune sera elicited by H5N1 vaccination, especially the adjuvanted high dose (30μg +), conferred full protection against a lethal *in vivo* challenge.

### Neutralizing Antibody and Neuraminidase Inhibiting Antibody Titers Act as Correlates of Protection

Our final aim was to study which of the H5N1 vaccine elicited antibody responses correlated with *in vivo* protection. We included the widely used hemagglutination inhibition (HI) titer, neutralizing antibody titer measured in pseudotype-based assay (PN titer), hemagglutinin stalk specific antibody level (HA stalk IgG) and neuraminidase inhibition (NI) titer as candidates for correlates of protection. As the Fc receptors involved in ADCC are different between humans and mice, the ADCC inducing antibody titer was not included in analyses.

**Figure 5:**
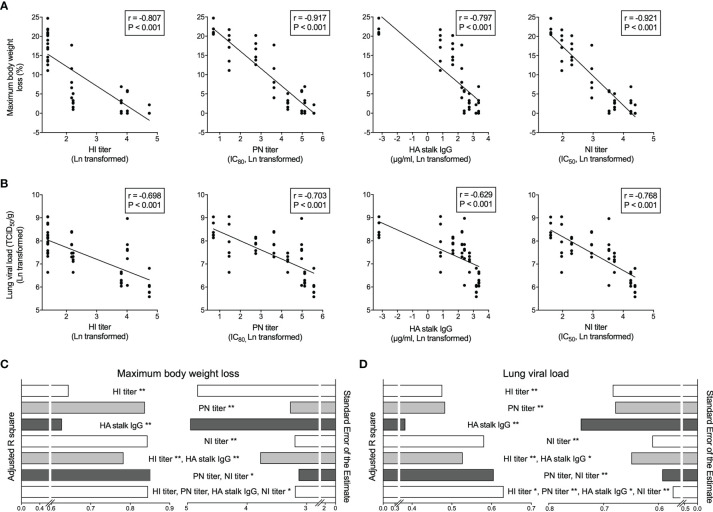
Vaccine elicited HA and NA specific antibodies predict *in vivo* protection after challenge. **(A, B)** Hemagglutination inhibition antibodies (HI titer, far left), HA specific neutralizing antibodies (PN titer, central left), total HA stalk specific antibodies (HA stalk IgG, central right), and neuraminidase enzymatic activity inhibiting antibodies (NI titer, far right) significantly and inversely correlate with the maximum body weight loss **(A)** and lung viral load **(B)** in mice after NIBRG-14 virus challenge. Each symbol represents one mouse **(A, B)**. The linear fitting curve is plotted as line. Pearson r and P values are noted in each correlation. **(C, D)** Antibody parameters including HI titer, PN titer, HA stalk IgG and NI titer results were used to predict mice maximum body weight loss **(C)** and lung viral load **(D)** in uni- and multiple linear regression models. Adjusted R square (left half) and Standard Error of the Estimate (right half) are shown to indicate the goodness of different models explaining dependent variables. In each model, predictors contributing significantly are noted. *P < 0.05, **P < 0.01. For detailed model summary and coefficients, see **Supplementary Tables 2–3**. Antibody parameters from each individual subject in clinical trial were put together in silico the same way as sera were combined to make group-and-time-point-wise pooled human sera for mice passive transfer. Geometric mean of antibody parameter was calculated, Ln transformed and centered before being used as independent variables. Maximum body weight loss and Ln-transformed lung viral load were used as dependent variables in regression analyses in SPSS 25.

Importantly, we observed inverse correlations between the antibody levels in the pooled human sera that mice received by passive transfer and murine body weight loss, as well as lung viral load after challenge (**Figures 5A, B**). Of note, the NI titer had the closest relationship with both murine body weight loss and lung viral load, followed by PN titer. Whilst the HI titer and HA stalk IgG level showed lower degrees of correlation with *in vivo* protection, determined by the Peason’s r values in correlation analyses.

We further dissected the contribution of each antibody parameter to the predictions of *in vivo* protection. The adjusted R square and the standard error of the estimate in the multiple linear regression analyses were used to determine the level of fit in different prediction models. When predicting the maximum body weight loss, PN titer and NI titer alone or combined were among the best-fit models (**Figure 5C** and **Supplementary Table 2**). In the lung viral load predictions, NI titer alone, NI and PN titer combined, and all 4 antibody parameters together were the top 3 models (**Figure 5D** and **Supplementary Table 2**). Notably, NI titer contributed significantly to the top models predicting both body weight loss and lung viral load (**Figures 5C, D** and **Supplementary Table 3**). Together, our correlation and regression analyses on the *in vivo* protection provided by pooled human sera in murine challenge model cohesively demonstrated that PN and NI titers act as correlates of protection. Further study is needed to confirm the roles of PN and NI titers as correlates of protection in human infection scenario.

## Discussion

Since 1996, highly pathogenic avian influenza (HPAI) H5N1 have caused outbreaks in domestic and wild birds worldwide, as well as sporadic zoonotic-human transmissions (3, 26). The high mortality rate in confirmed human infections raised alarms about the global pandemic preparedness (1). Dozens of pre-pandemic H5N1 candidates have been developed, and clinical trials have been conducted with vaccines developed in various platforms (3, 5). Here, we investigated in-depth the multifaceted antibody responses after a virosomal H5N1 vaccine alone or with the Matrix M adjuvant. Our data showed that the adjuvanted H5N1 vaccines elicited potent vaccine-specific and broadly cross-neutralizing antibodies, as well as HA stalk and NA specific functional antibodies. The multifaceted antibody responses were found as early as 7 days after the first dose and were further boosted and maintained at high levels after the second dose. These antibodies provided full protection against H5N1 virus challenge in mice. Of note, the levels of neutralizing antibodies and NA inhibiting antibodies could predict *in vivo* protection against infection and disease progression.

Like other enveloped RNA virus, influenza viruses evolve continuously due to its error-prone replication and large host reservoirs. Newly emerged strains may thus escape existing immunity established from previous infection and vaccination. The HPAI H5N1 viruses have evolved into multiple clades and subclades. Therefore, vaccines capable of eliciting cross-protective immunity are highly desired. Previous studies on cross-neutralizing antibodies after H5N1 vaccines were mostly limited to clades 1 and 2 (8, 27–30). To our knowledge, we are the first to report vaccine-induced cross-neutralizing antibody responses against all H5 clades found from human infections, including (sub)clades 1, 2.1.3.2, 2.2.1, 2.3.2.1, 2.3.4 and 7. To our surprise, most of the subjects had low to moderate level of antibodies cross-reactive to multiple (sub)clades prior to vaccination. Importantly, the Matrix M adjuvanted virosomal H5N1 vaccine elicited potent neutralizing antibody responses against all 6 (sub)clades, as quickly as 7 days after the first vaccine dose. Similarly, rapid increases in functional non-neutralizing antibodies, such as HA stalk IgG, NA inhibiting and ADCC inducing antibodies, were observed after vaccination (**Figures 1**, **2**). In contrast, our early studies using HI, microneutralization and single radial hemolysis assays showed no measurable pre-existing antibodies; and 2 doses of vaccine were required for induction of low to moderate levels of antibodies that could cross neutralize clades 1 and 2 [**Supplementary Figure 1** and references (16–18)]. The detection of pre-existing antibodies in this study could be attributed to the higher sensitivity of pseudotype-based neutralization compared to the traditional assays used earlier. The protective potential of these pre-existing antibodies has not yet been fully elucidated, but our results from the sera passive transfer and viral challenge in mice indicate that efficient vaccines will still be needed for protection. The different magnitude and kinetics of the antibody response emphasize the necessity of implementing and harmonizing highly sensitive serological assays to fully understand the immune responses. For example, a more balanced distribution of surface glycoproteins and sensitive luminescence readout make pseudotype-based neutralization assay more sensitive, quantitative and better suited in detecting zoonotic virus specific antibodies (19, 31, 32).

Compared to the variable HA head domain, the HA stalk domain and NA are more conserved. Thus, functional antibodies against HA stalk and NA broaden vaccine responses against heterologous strains. Ellebedy et al. reported robust HA stalk antibody responses measured in ELISA after receiving inactivated H5N1 vaccine (7). Boudreau C. et al. demonstrated that the MF59-adjuvanted H5N1 vaccine elicited antibodies that stimulated robust neutrophil phagocytosis and complement activity (33). In our study, we observed potent increases of HA stalk specific IgGs and ADCC inducing antibodies, which peaked after priming with no further boost after the second vaccine dose. By contrast, cross-neutralizing and NA inhibiting antibodies were significantly elicited after both the priming and boosting dose, especially in the low and intermedium adjuvanted (1.5μg + and 7.5μg +) groups (**Figures 1**, **2**).

Adjuvants enhance vaccine immunogenicity, especially in the absence of pre-existing immune memory. The Matrix M has been tested in vaccines against malaria and is currently used in the NVX-CoV2373 vaccine against COVID-19 (34–38). Here we demonstrated that the Matrix M adjuvanted H5N1 influenza vaccine induced potent increases of broadly cross-neutralizing antibodies, as well as HA stalk and NA specific non-neutralizing antibodies. The adjuvanted 7.5μg + and 30μg + vaccines induced significantly higher titers and better breadth of antibodies as compared to the non-adjuvanted 30μg - group. More importantly, days 21 and 42 post-vaccination sera from 30μg + group provided full protection in mice against both disease progress and viral infection (**Figure 4**).

The controlled human influenza virus infection model and vaccine field studies are useful in assessing the vaccine effectiveness and establishing correlates of protection (39, 40). Unfortunately, it is difficult to conduct such studies with HPAI H5N1 viruses due to the high morbidity and mortality rate associated. We therefore transferred human sera into naïve mice before an *in vivo* challenge to study relevant correlates of protection. Other studies applying human sera transfer and mice challenge have shown that HA stalk specific antibodies predict *in vivo* protection against heterologous virus challenge (41, 42). Here, our results demonstrated that PN and NI titers had closer correlations with *in vivo* protection against a homologous virus challenge, as compared to HI titer or HA stalk specific IgG (**Figure 5**). These results are in agreement with other human challenge (43) and cohort studies (44–46). A caveat here is that our multiple linear regression analyses suffer from inherited collinearity issues due to the similar kinetics of vaccine-elicited antibody responses. Therefore, the coefficients of each predictor may be subject to variance inflation (**Supplementary Table 3**). Nevertheless, the overall level of predictions should hold accurate (**Figures 5C, D** and **Supplementary Table 2**).

In this study, we assessed the kinetics, magnitude and *in vivo* protection efficacy of the multifaceted antibody responses after the adjuvanted virosomal H5N1 vaccines. This vaccine was developed in preparation for a potential H5N1 pandemic. Nevertheless, lessons learnt from H5N1 vaccine development could help preparation for the ongoing and future pandemics: 1) Highly sensitive assays help better understand the breadth of cross-reactive immune responses after infection and vaccination. 2) Adjuvants allow vaccine dose sparing and enhance both the magnitude and breadth of responses against vaccine strain and heterologous variants.

## Data Availability Statement

The raw data supporting the conclusions of this article will be made available by the authors, without undue reservation.

## Ethics Statement

The studies involving human participants were reviewed and approved by The Regional Committee for Medical Research Ethics, Northern Norway and the Norwegian Medicines Agency. The patients/participants provided their written informed consent to participate in this study. The animal study was reviewed and approved by Norwegian Food Safety Authority.

## Author Contributions

FZ and RC conceived the project. FZ, GG, and RC designed the experiments. RC and GP conducted the clinical study and collected samples. FZ, LH, and GG conducted the experiments. FZ analyzed the data. FZ, LH, GP, GG, and RC prepared and edited the manuscript. All authors contributed to the article and approved the submitted version.

## Funding

This study received intramural funding from the Influenza Center at the University of Bergen and Haukeland University Hospital. The Influenza Center is funded by the University of Bergen, Ministry of Health and Care Services, the Trond Mohn Foundation (TMS2020TMT05), the European Union (EU IMI115672 FLUCOP, H2020 874866 INCENTIVE, H2020 101037867 VACCELERATE, EU IMI 101007799 Inno4Vac) and Nanomedicines Flunanoair (ERA-NETet EuroNanoMed2, JTC2016), and the Research Council of Norway GLOBVAC program (284930).

## Conflict of Interest

The authors declare that the research was conducted in the absence of any commercial or financial relationships that could be construed as a potential conflict of interest.

## Publisher’s Note

All claims expressed in this article are solely those of the authors and do not necessarily represent those of their affiliated organizations, or those of the publisher, the editors and the reviewers. Any product that may be evaluated in this article, or claim that may be made by its manufacturer, is not guaranteed or endorsed by the publisher.
